# Impact of ultra-shear technology on quality attributes of model dairy-pea protein dispersions with different fat levels

**DOI:** 10.1016/j.crfs.2023.100439

**Published:** 2023-01-11

**Authors:** Jerish Joyner Janahar, V.M. Balasubramaniam, Rafael Jiménez-Flores, Osvaldo H. Campanella, Bhavesh Patel, Joana Ortega-Anaya

**Affiliations:** a Department of Food Science and Technology, The Ohio State University, Columbus, OH, 43210, USA; b Department of Food Agricultural and Biological Engineering, The Ohio State University, Columbus, OH, 43210, USA

**Keywords:** Dairy, Pea protein, Fat, High pressure, Shear, Rheological properties, Stability

## Abstract

This study investigated the impact of ultra-shear technology (UST) processing on dairy-pea protein dispersions with different fat levels. Raw milk, skim milk, and cream, as well as model dispersions with combinations of dairy products and pea protein (i.e., raw milk with pea protein, skim milk with pea protein, and cream with pea protein) were employed as test samples. UST experiments were conducted at a pressure of 400 MPa and 70 °C shear valve exit temperature. The UST treatment increased the viscosity of the dispersions and the increases depended on the fat level. Dairy-pea protein dispersions from raw milk and skim milk were shear thinning and mathematically described by the power-law model defined by the consistency coefficient, *K* (Pa·s^n^) and the flow behavior index, *n*. UST treated cream + pea protein dispersions produced structures with gel-like characteristics. Microstructure and particle size analysis determined by laser scanning microscope revealed a reduction in particle size after UST treatment in raw milk + pea protein and skim milk + pea protein dispersions up to 7.55 and 8.30 μm, respectively. In contrast, the particle mean diameter of cream + pea protein dispersions increased up to 77.20 μm after the UST treatment. Thus, the effect of UST on the particle size and rheological behavior of the dispersions depended on the fat level. UST-treated dispersions were stable with no visible phase separation or sedimentation upon centrifugation at 4000×*g* for 30 min (4 °C). Heat treatment and freeze–thaw treatment of UST-treated samples showed stable blends immediately after the treatments, but subsequent centrifugation showed solid separation. Results from the study suggest that UST is a potential technology to produce stable dairy + pea protein liquids foods with different rheological characteristics for diverse applications.

## Highlights

• UST modified the viscosity of pea-dairy protein dispersions depending on fat level.
• UST increased or decreased the particle size in dispersions based on the fat level.
• UST-treated dispersions were stable, with no separation or sedimentation.
• UST may help create various clean-label plant-dairy protein blended liquid foods.

## Graphical abstract

**Figure:**
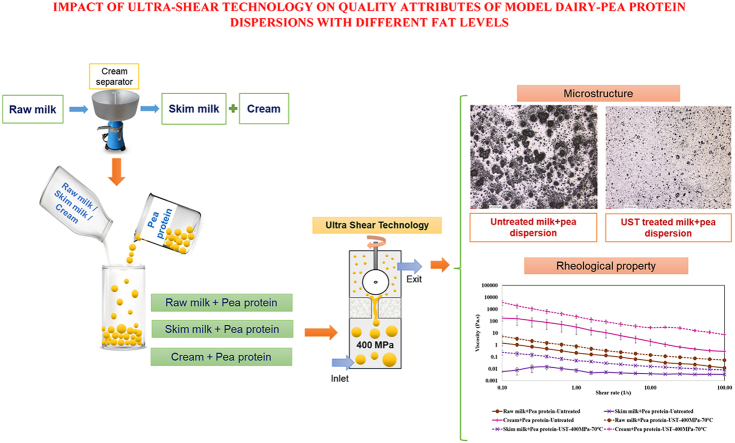

## Abbreviations

UST: Ultra-Shear Technology
HPP: High-Pressure Processing
HPH: High-Pressure Homogenization
TP: Thermal Processing
HTST: High Temperature Short Time
UP: Ultra Pasteurization

## 1 Introduction

Increasing consumer awareness on health-promoting foods has created a demand for novel foods containing nutritious proteins and lipids from different sources. In addition, the increasing world population is demanding the use of sustainable protein sources. Dairy proteins are widely consumed and confer several nutritional benefits. Pea protein is a commonly used ingredient in plant-based foods due to its high nutritional content, affordability, low allergenicity and sustainability (Lam et al., 2018). In recent years, pea proteins have been regarded as suitable ingredients to replace or complement dairy proteins in beverages (Boukid et al., 2021). Demand for plant protein drinks is rising due to their diverse nutritional profiles, perceived sustainability benefits, and difficulties in generating enough animal proteins to meet the dietary needs of the growing global population in a sustainable manner. Blending pea and dairy proteins would be an economical way to provide nutritious and sustainable protein-based foods. However, inclusion of pea protein in protein-based beverages is challenging, due to the low dispersibility of pea proteins in the presence of a lipid phase which may cause separation and sedimentation of pea protein particles in the beverage continuous phase.

Mixtures of dairy and plant proteins such as whey protein-pea protein isolates (Ho et al., 2018; Hinderink et al., 2019), and sodium caseinate-soy protein (Ji et al., 2015) have been used as emulsifying ingredients to stabilize oil-based emulsions such as soy, canola, and sunflower oils. Under these circumstances, the protein contents were low because proteins were used as minor ingredients or as aids in processing. On the other hand, when dairy and plant proteins are used as the primary ingredients, food scientists and engineers face multiple challenges including sedimentation of plant proteins and separation of milk fat. This results in unacceptable non-homogenous products. One practical solution to overcome this problem is to use additives that work as emulsifiers and/or stabilizers or thickening agents. However, with a consumer interest in clean-label liquid foods, food processors are interested in developing technological solutions to attain stable and homogeneous blends without the use of chemical additives.

High-pressure homogenization (HPH) is a semi-continuous process that involves exposure of liquid food to high pressure of about 400 MPa and sudden depressurization by passing through a tiny gap in a shear valve (Balasubramaniam, 2021). The instant pressure drop leads to the conversion of pressure energy to kinetic energy, which is transformed to shear, turbulence, cavitation, high velocity collisions, and heat. These phenomena enable useful functions such as particle size modification, emulsification, mixing, microbial and enzyme inactivation, and in general significant changes in dispersion or emulsion characteristics. Recently, ultra-shear technology (UST) was developed to address some of the limitations of HPH. The UST uses a self-throttling valve which uses a dynamic force control mechanism. Valve gap is adjustable according to the pressure and composition of the fluid, thereby minimizing clogging of the shear valve. UST employs fluidic isolator to decouple the liquid food from physical contact with the pump providing the high pressure. The self-throttling valve maximizes fluid velocity and minimizes gap distance to achieve shear rate up to 10^7^ s^−1^ (Smejkal et al., 2021).

Several studies have used high pressure- and shear-based mechanisms to stabilize dairy products, inactivate bacteria and enzymes, and promote structural changes in milk proteins (Pereda et al., 2007; Roach and Harte, 2008; Martínez-Monteagudo et al., 2017; Janahar et al., 2021). In a previous study, we have demonstrated the feasibility of using UST for blending dairy products-plant proteins to produce protein rich beverages with different protein concentrations (Janahar et al., 2022). Unlike high-pressure processing (HPP) or thermal processing (TP), UST treatment reduced the particle size in dispersions with lower protein concentrations, however, aggregation of particles was observed above certain concentrations. Similarly, UST treatment altered the rheological characteristics of the dairy products-pea protein dispersions and created homogenous liquids, stable emulsions, and gel structures depending on the treatment intensity, the protein nature and its content.

The amount of fat is associated with perceived creaminess and is positively related to the sensorial product liking in dairy products such as sour cream (Jervis et al., 2014; McCarthy et al., 2017). Moreover, the fat content of a food is associated with the apparent viscosity (Akhtar et al., 2005) and the rheology of the dispersions (Chojnicka-Paszun et al., 2012). However, very limited information is available on the influence of variations in fat level on the UST-treated dairy-plant protein liquid dispersions.

The objectives of this study were to investigate the feasibility of UST to produce dairy-pea protein dispersions with different fat levels and to control the resulting viscosity without the use additives. The influence of UST on the quality attributes were characterized in terms of viscoelastic characteristics, microstructure, particle size, soluble protein content, pH, zeta potential, and stability.

## 2 Materials and methods

### 2.1 Preparation of milk with different fat levels

Raw cow milk was obtained from The Ohio State University (OSU) Dairy Farm, Columbus, Ohio and transported at ≤4 °C to the OSU Emerging Food Process Technology pilot plant within 30 min. The raw milk (4.9 ± 0.3% fat, 13.5 ± 0.14% total solids) was heated to 45–55 °C and separated into cream (50.2 ± 1.1% fat, 55.2 ± 3.5% total solids) and skim milk (0.34 ± 0.03% fat, 10.29 ± 0.04% total solids) using an Elecrem1 cream separator (Elecrem, Vanves, France). The samples were stored at ≤4 °C for a maximum of 24 h before further treatments and analysis.

### 2.2 Preparation of model pea-milk protein dispersions with different fat levels

Milk with different fat levels viz., raw milk, skim milk, and cream were collected in 500 mL beakers. For each 100 mL of raw milk, skim milk, or cream, about 4 g of pea protein (80% protein) was added to have 1:1 protein ratio in the model dairy-pea protein systems. Thus, three model dispersions with different fat levels were prepared, namely, i) raw milk + pea protein, ii) skim milk + pea protein, iii) cream + pea protein. The dispersions were allowed to hydrate for 3 h prior to processing.

### 2.3 Ultra-shear technology treatment

A custom-fabricated benchtop UST laboratory tester (PBI, MA, USA) was used for the UST treatment (Janahar et al., 2021). During a typical experiment, the dairy-pea protein dispersion at 13 ± 2 °C was fed into the UST pressure chamber, and pressurized. The dispersion was subsequently depressurized by passing through a shear valve at exit temperature of 70 °C and collected aseptically. This cycle was repeated to collect desired volume of treated sample for further analysis. The time interval between cycles can be adjusted, and an interval of ∼10–15s was used in the study.

**Fig. 1:**
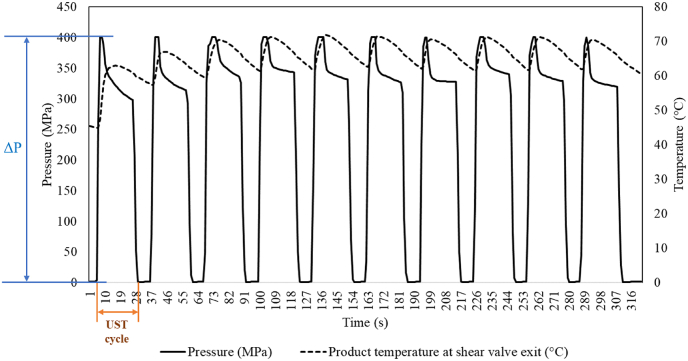
Pressure-temperature history of dispersions during UST treatment at 400 MPa and temperature of 70 °C at shear valve exit.

At the start of the experiment, samples from first two cycles were discarded, as the equipment components (initially at ambient temperature), were not at the target process temperature. Samples were collected from the 3^rd^ process run (cycle) onwards to ensure that the sample received the target temperature (70 °C) at the shear valve exit (Fig. 1). The UST treated samples were immediately cooled down to <5 °C by placing them in an ice-water bath. The flow rate of the dispersion through the UST shear valve was 0.0013 ± 0.0006 kg/s. Pressure and temperature data were recorded by a data acquisition system (PBI, MA, USA).

### 2.4 Analysis

After a resting time of about 12 h following the UST treatments, the quality attributes of samples were evaluated using rheological measurements, microstructure, particle size, soluble protein, pH, zeta potential, and stability.

### 2.5 Rheological measurements

The rheological characterization of samples was performed in a Discovery-HR3 rheometer (TA instruments, New Castle, DE, USA) with a 40 mm parallel plate geometry at 25 °C temperature and a gap of 1000 μm.

#### 2.5.1 Frequency sweep

First, strain sweep measurements were carried out between 0.1 and 1000% strain at a frequency of 1 Hz to determine the linear viscoelastic range. A strain of 1% was selected in the linear viscoelastic region and a frequency sweep was performed with frequency ranging from 0.1 to 100 rad/s. The elastic or storage modulus (G′) and viscous or loss modulus (G″) were obtained as a function of the angular frequency (ω), which vary with the viscoelastic properties of the samples and the physical stability of the blends. Frequency sweep measurements were performed only for UST-treated and untreated dispersions including pea protein, since viscoelastic properties were observed on these samples during preliminary analysis.

#### 2.5.2 Flow sweep

Steady state shear flow measurements were carried out at increasing shear rates from 0.1 to 100 s^−1^ to measure the shear viscosities of samples.

Rheological data was analyzed using TRIOS software (TA Instruments, New Castle, DE, USA). Measurements were made in triplicate and average values are reported. To characterize the flow behavior of samples, the Newtonian or power law models were used. The flow behavior index (*n*, dimensionless) and consistency coefficient (*K*, Pa·s^*n*^) values were computed by fitting the rheological data to the power law model (Eq. (1)).

(1) $$\tau =K{\dot{\gamma }}^{n}$$

Where, *τ* is the shear stress (Pa) and $\dot{\gamma }$ is the shear rate (s^−1^). When *n* = 1, *n* < 1, and *n* > 1 the fluids are called Newtonian, pseudoplastic, and dilatant, respectively. The correlation coefficient (R) and standard error of estimates (SEE) were determined using Eqs. (2), (3)).

(2) $$R=\frac{\sum \left(x-\bar{x}\right)\;\left(y-\bar{y}\right)}{\sqrt{{\sum \left(x-\bar{x}\right)}^{2}{\sum \left(y-\bar{y}\right)}^{2}}\;}$$

(3) $$SEE=\sqrt{\frac{1}{\left(n-2\right)}\left[{\sum \left(y-\bar{y}\right)}^{2}-\frac{{\left[\sum \left(x-\bar{x}\right)\left(y-\bar{y}\right)\right]}^{2}}{{\sum \left(x-\bar{x}\right)}^{2}}\;\right]}$$

Where, *x* is the observed value, *y* is the estimated value, $\bar{x}$ and ȳ are the means of observed and estimated values, respectively, and n is the count of the total number of (*x,y*) pairs.

### 2.6 Microstructure and particle size characterization

To obtain the microstructure, well-mixed samples (5 μL) were carefully placed onto a glass slide, spread as a thin layer, and allowed to air dry at room temperature (20–22 °C) for 12 h. Then, the microstructure was observed at 10 × magnification, and 2D and 3D (laser and optical) images were obtained by a VK-X200 series non-contact 3D laser scanning microscope (Keyence, Osaka, Japan). VK-Analyzer v3.3.0.0 software (Keyence, Osaka, Japan) was used to analyze the images.

To characterize the particle sizes of protein and/or fat aggregates, at least three images (area: 1426 × 1069 μm) were analyzed using VK-Analyzer software (Keyence v3.3.0.0) and the mean diameter and average height of particles were determined. To measure the mean diameter, the contour of each fat-protein particle was assumed circular and fitted using the 3-point diameter function. At least 20 measurements were obtained, and mean values are reported. The measurement of average height of particles was performed on the 3D images based on confocal profiling in the laser microscope.

#### 2.6.1 Soluble protein analysis

The protein solubilities of the untreated and UST-treated raw milk + pea protein, skim milk + pea protein, and cream + pea protein dispersions were determined using bicinchoninic acid (BCA) assay kit (Pierce Biotechnology Inc., Rockford, IL). Briefly, the dispersions (5 mL) were centrifuged at 4000×*g* for 60 min at 4 °C. Protein in the initial sample and supernatant portion after centrifugation were determined using BCA assay (Smith et al., 1985) using Bovine serum albumin (BSA) as the protein standard. The soluble protein concentration in the supernatant was noted in mg/mL. Protein solubility (%) is given as the percent ratio of protein in the supernatant to the total protein in the initial sample before centrifugation.

The hydrodynamic diameter of the particles in the aqueous supernatant diluted with 5 mL of distilled water were measured by dynamic light scattering in a particle size analyzer (NanoBrook, ZetaPALS, Brookhaven, NY). For the measurement, 100 μL of the sample was added to 3 mL of distilled water in a clean cuvette. A particle refractive index of 0.133, viscosity of 0.890 mPa s, pH of 7.0, and backscattering angle of 173° was used in the particle size analyzer.

### 2.7 pH and zeta potential

The pH of samples was measured at 23 ± 2 °C using a benchtop pH meter (Mettler-Toledo, USA). For zeta potential measurements at this pH, samples were diluted with ultra-pure water in the ratio of 1:1000 and placed in a zeta potential analyzer (NanoBrook, ZetaPALS, Brookhaven, Holtsville, NY). The electrophoretic mobility of particles was measured using the phase analysis light scattering technique with a detection angle of 15° and the Smoluchowski model was used to convert the mobility data into zeta potential values.

### 2.8 Stability

The stability of the samples was determined in three different ways, namely, colloidal stability, heat stability, and freeze–thaw stability.

#### 2.8.1 Colloidal stability

The colloidal stability of the samples was analyzed using centrifugal forces to accelerate the occurrence of instability phenomena such as sedimentation or creaming. The method described by Baier et al. (2015) was followed with a slight modification. Five mL of each sample taken in graduated tube were centrifuged at 4000×*g* for 30 min at 4 °C in a Sorvall Legend XFR centrifuge (Thermo Scientific, Waltham, USA) and the volume fractions of separated milk fat, sediment, and serum were noted from the graduations in the tube.

The volume fractions were determined using the following equations:

(4) $$Volume\;of\;sediment\;\left(\%\right)=\left(\frac{Volume\;of\;sediment}{Total\;volume}\right)\times 100$$

(5) $$Volume\;of\;blend\;\left(\%\right)=\left(\frac{Volume\;of\;blend}{Total\;volume}\right)\times 100$$

(6) $$Volume\;of\;serum\;\left(\%\right)=\left(\frac{Volume\;of\;serum}{Total\;volume}\right)\times 100$$

(7) $$Volume\;of\;separated\;milk\;fat\;\left(\%\right)=\left(\frac{Volume\;of\;separated\;milk\;fat}{Total\;volume}\right)\times 100$$

Here, *sediment* refers to pea protein sediment in the dispersion, *serum* refers to milk/liquid portion with no separated fat or pea particles, and *blend* refers to the inseparable mix of milk fat + serum or milk fat/serum + pea protein.

It is important to assess the stability of UST-treated samples under various conditions experienced during storage/consumption such as heating or freeze–thaw cycles in households by the consumers before consumption. So, the following tests were carried out.

#### 2.8.2 Heat stability

While using UST-treated liquid foods, consumers may prefer heating before consumption. Thus, the product stability under such circumstances was evaluated. Various untreated and UST-treated samples were evaluated for thermal stability around boiling conditions ∼100 °C (97 ± 3 °C) for 10 min using a temperature controlled hot water bath. The come-up time for the temperature was about 4 min. Then, the samples were centrifuged at 4000×*g* for 30 min at 4 °C in the Sorvall Legend XFR centrifuge (Thermo Scientific, Waltham, USA). The different volume fractions were calculated before and after centrifugation using equations (4), (5), (6), (7)).

#### 2.8.3 Freeze–thaw stability

To determine the freeze–thaw stability of the UST-treated samples, the samples were placed in a freezer (−20 °C) for 24 h and subsequently thawed under refrigerated conditions (≤4 °C) for 12 h. Then, the samples were centrifuged at 4000×*g* for 30 min at 4 °C in Sorvall Legend XFR centrifuge (Thermo Scientific, Waltham, USA). The different volume fractions were calculated before and after centrifugation using equations (4), (5), (6), (7)).

### 2.9 Statistical analysis

All process runs and instrumental measurements were carried out in triplicate, unless mentioned otherwise, and results were reported as mean and standard deviation. To evaluate the influence of fat level, pea protein, UST treatment, and resulting interactions, a three-way ANOVA was performed using IBM SPSS Statistics 27 (IBM Corporation, Armonk, USA) software package. For comparison of means, Tukey's test was used. Statistical significance was considered as *P* ≤ 0.05.

## 3 Results and discussion

### 3.1 Pressure-thermal history of ultra-shear technology-treated samples

Fig. 1 illustrates the representative pressure-thermal history of UST treated liquid dispersions subjected to multiple UST cycles. During a typical UST cycle, the fluid temperature instantaneously increased at the exit of the shear valve due to heat of homogenization (Janahar et al., 2021). The instantaneous temperature rise could help to reduce the product’s prolonged thermal exposure (Balasubramaniam et al., 2023). The decreasing temperature trend (see Fig. 1) was as a result of intermittent fluid flow through shear valve between successive UST cycles.

The temperature of the product (T_p_) as it exits the shear valve can be estimated by the following relation.

(8) T_p_ ≈ T_i_ + δ*ΔP + λ*ΔP ± ΔT_h_

where, T_i_ is initial liquid temperature, δ is the heat of compression (°C/100 MPa), λ is the heat of homogenization (°C/100 MPa), and ΔT_h_ is the heat loss/gain with the environment. It is worth noting that, at the beginning of the experiments, equipment components (shear valve and connecting pipes among others) may not be at the target temperature, and with subsequent progression of UST cycles, the desired process temperature at shear valve exit was stabilized.

The δ values of water, juice, milk, and similar liquid foods was reported to be 3 °C/100 MPa at 25 °C and the δ values increase with product initial temperature. It is also worth to note that temperature increase due to δ is transient and product temperature returns close to initial value upon decompression.

Unlike δ, the theoretical maximum estimate for λ for water at 25 °C was estimated as 26.20 °C/100 MPa (Janahar et al., 2021). This temperature increase is irreversible. For dairy beverages, the experimentally determined λ values in laboratory, pilot scale equipment ranged between ∼15 and 20 °C per 100 MPa (Martínez-Monteagudo et al., 2017; Zamora et al., 2012; Hayes and Kelly, 2003). In the present study, the heat of homogenization of various samples ranged between 16.44 and 19.04 °C per 100 MPa, which was within the reported values.

### 3.2 Rheological measurements

#### 3.2.1 Frequency sweep

**Fig. 2:**
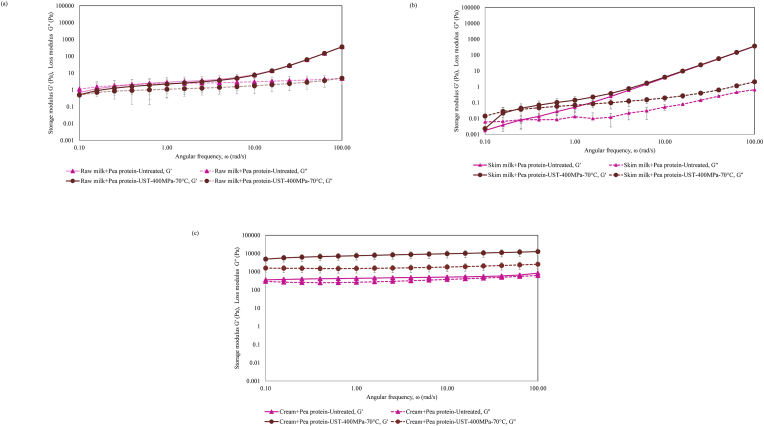
Frequency sweep analysis showing storage modulus, loss modulus of dairy-pea protein dispersions before and after UST treatment a) raw milk, b) skim milk, and c) cream.

The frequency sweep measurement provides information on the colloidal forces and the degree of particle–particle interactions involved in the rheological characteristics of the material (Aguilar-Zárate et al., 2019). The viscoelastic characteristics, namely storage modulus (G′) and loss modulus (G″) of raw milk, skim milk, and cream samples with added pea protein are shown in Fig. 2a-c. The G′ and G″ values of both untreated and UST-treated low-fat samples, namely raw milk and skim milk with added pea protein, showed a gradual increase with frequency (Fig. 2a-b). This type of behavior is typically indicative of a weakly viscoelastic liquid system. García- Ortega et al. (2021) stated that when the G′ is more dependent on frequency, the material is more fluid-like. Thus, the low-fat samples with added pea protein treated by UST in the study could potentially serve as dairy-plant protein blended drinks or sauces. The G′ and G″ of untreated and UST-treated high-fat, i.e., cream + pea protein samples were higher than the low-fat samples indicating the role of fat content in increasing the elasticity of the dispersions. The G′ of 70 °C UST-treated cream + pea protein samples were higher than untreated samples, which indicated the role of UST in creating stronger viscoelastic networks in samples (Fig. 2c). G′ > G″ for most of the frequencies, which reflected the strong elastic or gel-like characteristic of these samples. Further, the G′ and G″ of UST-treated high-fat samples appeared to be independent of frequency, which demonstrated the textural strength and the structural stability of the samples. In materials with strong gel-like characteristics, the G′ is nearly independent of angular frequency (García-Ortega et al., 2021). Accordingly, Tanger et al. (2021) observed a lower frequency dependence and a higher G′ in a model milk dessert where 50% of fat was replaced by pea protein and reported more gel-like characteristics caused by more stabilizing interactions in these products. Additionally, researchers have elucidated the role of milk fat in rheological characteristics of dairy products. Barrantes et al. (1996) studied the rheological characteristics of yoghurt emulsified with skim milk powder (∼14% w/w) and containing anhydrous milk fat (AMF) or vegetable oils (olive, groundnut, sunflower and maize) at 1.5% (w/w) level. The yoghurt samples with AMF showed increased firmness and reduced whey separation. The authors attributed this to the higher interaction between the milk fat globules and the milk proteins in these samples than the vegetable oil samples. Xu et al. (2008) reported that the higher the yoghurt fat content (0–3.5%), the faster the onset of gelation and more rigid was the gel network. The fat globules can act as structural promoters and interact with protein matrix. In the present study, the interactions could be accelerated by the intense mechanical forces of pressure, shear, and/or temperature during UST treatment which lead to elaborate structure formation in the dispersions. In addition, UST induces denaturation or aggregation of globular proteins in dairy (Dumay et al., 2013) and plant (Floury et al., 2002) sources. Utsumi et al. (2017) observed gel-like formation in soy milk homogenized at pressures above 200 MPa and attributed this formation to disulfide bonding and non-covalent bonds such as hydrophobic interaction and ionic and hydrogen bonding. Shear discharge associated with high pressure also causes hydrophobic reaggregation of fat globules (Tran et al., 2018). Thus, in the present study, the effects of pressure, temperature, and shear during UST on the milk fat, dairy, and pea proteins and the resulting interactions could be responsible for the gel network in blends.

#### 3.2.2 Flow sweep

**Fig. 3:**
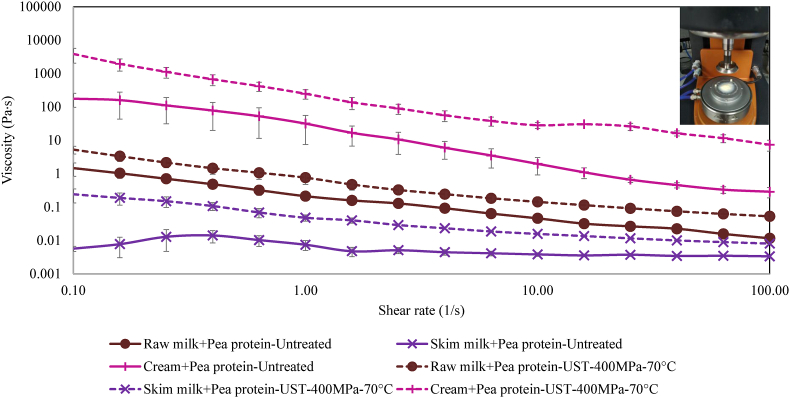
Viscosity as a function of the shear rate for dairy-pea protein dispersions before and after UST treatment. (Inset figure shows coagulation of fat globules in high-fat dispersion at high shear rate).

The viscosities of the dispersions with added pea protein at shear rates of 0.1–100 s^−1^ are shown in Fig. 3. The viscosities increased with increasing fat level. Further, the UST treatment increased the viscosities of the samples. The high pressure-associated shear during UST treatment created collision of fat-fat and fat-protein particles resulting in molecular entanglements that increased the viscosity. Furthermore, the UST process temperature could cause partial denaturation of proteins in samples, since the whey proteins in milk and pea proteins have initial denaturation temperatures around 70 °C (Lee, 1992; Mession et al., 2013). Protein denaturation and aggregation could lead to increase in viscosity. This observation was consistent with our previous research where we reported increase in viscosity of 2% (fat) milk dispersion with varying concentration of pea protein after 70 °C UST treatment (Janahar et al., 2022). Increase in milk viscosity due to aggregates formed by whey protein denaturation in high-temperature short-time (HTST) or ultra-pasteurized (UP) milk has been reported by Li et al. (2018). Denatured whey protein would have increased interactions with each other, and other components such as caseins, to form large complexes (Morales et al., 2000; Li et al., 2018). For cream and cream + pea samples, the viscosity increased at shear rates over ∼10 s^−1^. This increase could be attributed to the clustering of the individual fat globules at high-shear rates, as shown in Fig. 3 inset. Therefore, these data were excluded for further analysis.

#### 3.2.3 Rheological characteristics of milk with varying fat level

Raw milk and skim milk samples followed a Newtonian behavior (data not shown). The viscosities of untreated raw milk and skim milk were 2.8 mPa s and 1.7 mPa s and the viscosities increased to 7.1 mPa s and 5.3 mPa s, respectively, after 70 °C UST treatment. Similarly, UST treatment significantly increased the viscosity of the cream (untreated: *K* – 1.71 Pa·s^*n*^; UST-treated: *K* – 77.34 Pa·s^*n*^). This increase could be attributed to the tiny and dispersed state of fat and whey protein denaturation after UST treatment. These results agreed with our earlier study, where raw milk samples after 65 °C UST treatment showed higher denaturation of whey proteins and corresponding increase in the viscosity as compared to samples treated at relatively lesser temperature (35 °C) in UST (Janahar et al., 2021).

**Fig. 4:**
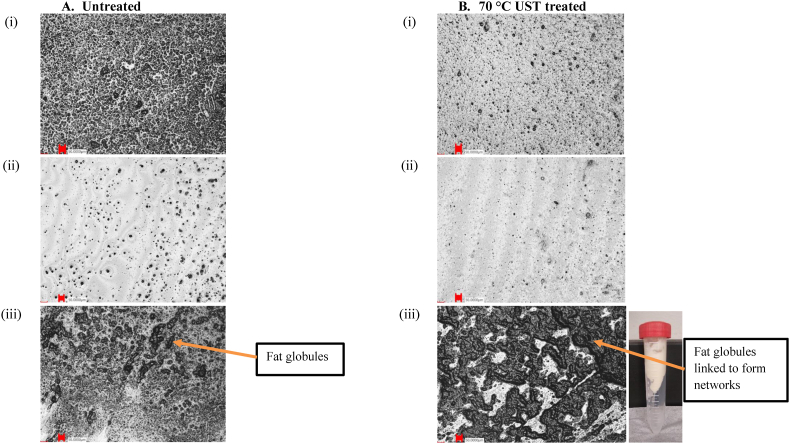
LSM images at 10 × magnification showing the microstructure of (A) untreated and (B) 70 °C UST-treated samples of (i) raw milk, (ii) skim milk, and (iii) cream ( - scale bar is 50 μm). The centrifuge tube (B - iii) shows UST-treated cream sample of high viscosity.

The behavior of cream with a high-fat content has been attributed to the cold agglutination phenomena in which immunoglobulins adhere to fat globules and agglomerate together into large floccules at temperatures below 35 °C (Vliet and Walstra, 1980; Vélez-Ruiz et al., 1997). The agglutination mechanism for immunoglobulin-fat complex formation could be deactivated at temperatures between 62 and 81 °C, which causes the milk fat globules to strongly interact with each other through milk fat globule membrane proteins or heat-denatured whey protein or destabilization of the casein micelles, resulting in a hard cream layer (Hansen et al., 2019). In addition to temperature, the high pressure and shear during UST could enhance the interactions of fat globules. The representative image showing the higher consistency of the UST-treated cream sample is shown in Fig. 4B(iii).

#### 3.2.4 Rheological behavior of dispersions with pea protein

For the dairy dispersions with added pea protein, the flow curves (shear stress versus shear rate) were well described by the power law model with high correlation coefficients (R ≥ 0.98) (Table 1). The *n* of raw or skim milk samples with pea protein samples (with or without UST treatment) were less than 1, which evidenced pseudoplastic (shear thinning) behavior of samples. Earlier studies have reported shear-thinning behavior in emulsions treated by pressure-associated shear mechanism (Floury et al., 2002; Song et al., 2013). A colloidal product characterized by a low *n* would exhibit significant shear thinning behavior, possessing high viscosity at low shear rates and low viscosity at high shear rates, which can sensorily result in good mouth feel (Marcotte et al., 2001). Therefore, UST can be used for developing novel food beverages and substitutes with desirable and unique sensory characteristics. For raw milk and skim milk dispersions with pea protein, *K* of UST-treated raw milk + pea protein, and skim milk + pea protein increased by 226.2% and 55.6%, as compared to the respective untreated dispersions (Table 1).

**Table 1:** Power law model parameters of dairy dispersions with pea protein.

S.No.	Sample	Untreated				UST-400 MPa-70 °C			
		*K* (Pa·s^*n*^)	*n*	R	SEE	*K* (Pa·s^*n*^)	*n*	R	SEE
1.	Raw milk + Pea protein	0.42	0.35	0.99	0.032	1.37	0.41	0.98	0.067
2.	Skim milk + Pea protein	0.09	0.92	1.00	0.024	0.14	0.58	0.99	0.052
3.	Cream + Pea protein	a	a			a	a		

a *K* and *n* values were indeterminant, as untreated and treated cream + pea protein samples exhibited decreasing shear stress with increasing shear rate within the experimental conditions. This could be attributed to potential slippage and gel-solid structure of these samples.

For the high-fat cream + pea protein samples, viscoelastic results and flow tests indicated that the samples have a more gel characteristics and did not exhibit shear thinning or thickening behavior of liquids. The estimated power law model parameters for both untreated cream + pea protein (*K* = 24.92 Pa·s^*n*^, *n* = −0.22) and UST treated (*K* = 234.75 Pa·s^*n*^, *n* = −0.11) samples showed negative *n* values. These samples were physical gels with a high strength but a weak structure which did not regain its structure upon shearing. Fat molecules might be deflocculated and/or re-flocculated during the measurements. So, we choose not to report the results of these samples, and indicate as indeterminant in Table 1. The pressure-associated shear action in UST might have created interactions of fat and protein molecules that promoted increase in dispersions’ viscosity. For these samples their viscoelastic characterization using small strain oscillatory shear tests could be more relevant.

Further, the processing conditions involving combination of high pressure, shear, and temperature cause unfolding of proteins, which lead to increased water-binding capacity and swelling of proteins (Floury et al., 2002; Xiu et al., 2011). Thus, the increase of *K* could also be associated with increased water-binding capacity. Use of HPH to increase water-binding capacity and gel firmness has been reported in different products such as acid-coagulated yoghurts (Serra et al., 2009), fresh cheeses (Zamora et al., 2011), and yoghurts prepared from soymilk (Ferragut et al., 2009). Tanger et al. (2021) observed that incorporation of pea protein microparticles led to an increase in hardness, adhesiveness, and creaminess in the fat-reduced model milk dessert. Thus, the ability of the UST to alter the rheological properties of dispersions could also be utilized in development of fat-reduced products such as low-fat ice cream, salad dressings, yoghurt, sauces, and others.

### 3.3 Microstructure and particle size

#### 3.3.1 Microstructure

**Fig. 5:**
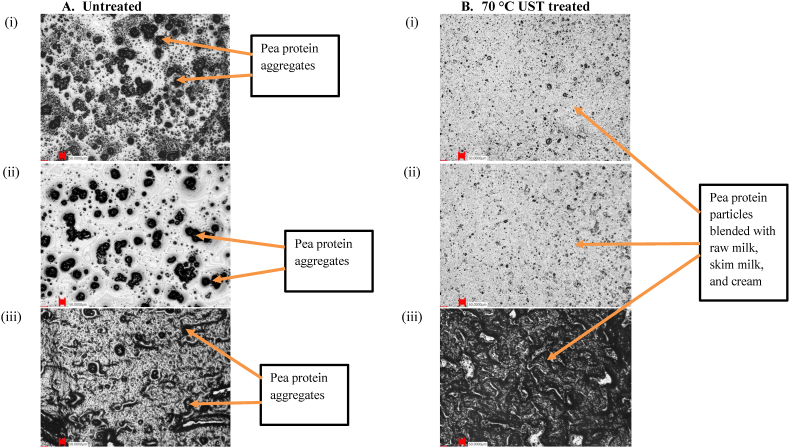
LSM images at 10 × magnification showing the microstructure of (A) untreated and (B) 70 °C UST-treated samples of (i) Raw milk + Pea protein, (ii) Skim milk + Pea protein, and (iii) Cream + Pea protein dispersions ( - scale bar is 50 μm).

The microstructures of samples with different fat levels and the effect of UST treatment on the microstructures are presented in Fig. 4, Fig. 5. Fig. 4A (i), (ii), and (iii) shows raw milk, skim milk, and cream, respectively. The large black particles represent fat globules, which shows that they are more densely arranged with the increase of fat content in the dispersion. Comparison of Fig. 4A and B(i) & (ii) reveals that the 70 °C UST treatment clearly reduced the particle sizes and created a homogenous dispersion. During UST treatment, the passage of larger particles through the tiny gap at high-shear conditions causes reduction of particle size through physical rupture. Additionally, the instant pressure drop across the valve generates a variety of phenomena such as shear, turbulence, and cavitation. This promotes particle collisions leading to particle size reduction when the internal resistance or strength of the particles is insufficient to sustain these forces. In low-fat (raw and skim milk) samples, UST treatment led to particle size reduction. In contrast, in high-fat (cream) samples, the 70 °C UST treatment appeared to increase the particle size [Fig. 4A&B-(iii)]. This increase might be because the UST process promoted the aggregation of small fat globules to form networks of fat.

Interestingly, a comparison of Fig. 4A and B(iii) reveals that after UST treatment, the serum (i.e., the liquid left after removal of fat globules from cream) is entrapped in the fat network. Furthermore, due to the strong mechanical conditions and high temperatures, the oil-in-water type emulsion in the untreated cream might be converted into a water-in-oil type emulsion (i.e., phase inversion) due to UST treatment. The high pressure-shear application might break the milk fat globules and cause liquid fat to expel out of the fat globules (Rønholt et al., 2013). The fat globules create networks with other fat and protein molecules due to UST treatment and the networks entrap the serum portion. This effect also resulted in increased viscosity of these samples, as discussed in section 3.2.

In Fig. 5A (i), (ii), and (iii) showing raw milk + pea protein, skim milk + pea protein, and cream + pea protein, respectively, the large black and nearly circular-shaped pea protein aggregates can be seen embedded in the dairy matrices. Fig. 5A–B, (i) and (ii) show that the UST treatment reduced the milk and pea aggregate sizes and made the blend homogenous. Fig. 5A and B (iii) illustrate that the UST treatment promoted homogenous cream + pea protein blends by aiding suspension of pea protein in the fat globules in cream. Like UST-treated cream samples, the cream + pea protein samples treated by UST showed visible linkages of protein and fat resulting in larger particle size. The high pressure, shear, and temperature led to particle size modification and promoted molecular interactions between pea protein, milk protein, and milk fat, which led to dairy-pea protein blends. The dense blends of UST-treated cream + pea blends (Fig. 5B–iii) also corroborate the higher viscosity and gel characteristic observed for these samples (Table 1).

#### 3.3.2 Particle size

The particle size parameters namely mean diameter and average height of the protein and/or fat aggregates in the dispersions are shown in Table 2. The fat level, pea protein, UST treatment and their interactions had significant effect (*P* ≤ 0.01) on the mean diameter and average height of the particles (Table 3). The mean diameter of untreated dispersions with inclusion of pea protein was significantly higher (*P* < 0.05) than dispersions without pea protein. The mean diameter of UST-treated samples with pea protein were significantly smaller (*P* < 0.05) than untreated samples, except for cream samples. The data supported the observation in 10.13039/501100009542LSM images of microstructures discussed in section 3.3.1. Several studies have reported reduction of particle size by pressure-associated shear treatment (Floury et al., 2004; Cortés-Muñoz et al., 2009; Song et al., 2013; Janahar et al., 2021). The particle size reduction could increase the number of particles and increase the probability of particle-particle interactions during application of pressure-associated shear (Dumay et al., 2013). The fat particles might be broken into small sizes and the pea protein might serve as a surface-active agent to cover the new fat interface and stabilize the blend.

**Table 2:** Particle size parameters, pH, and zeta potential of samples^a^.

Treatment^b^	Dairy source with different fat level	Mean diameter (μm)		Average height (μm)		pH		Zeta potential (mV)	
		Without pea protein	With pea protein	Without pea protein	With pea protein	Without pea protein	With pea protein	Without pea protein	With pea protein
Untreated	Raw milk	5.96^a^ ± 0.88	49.62^c^ ± 2.43	42.15^a^ ± 7.34	70.78^c^ ± 3.48	6.70^a^ ± 0.05	6.74^a^ ± 0.03	−53.66^ab^ ± 0.68	−54.68^ab^ ± 1.71
	Skim milk	5.94^a^ ± 1.22	51.76^c^ ± 4.51	14.79^b^ ± 4.49	73.53^c^ ± 4.49	6.73^a^ ± 0.03	6.74^a^ ± 0.01	−54.63^ab^ ± 1.50	−49.17^b^ ± 4.99
	Cream	27.39^b^ ± 2.29	48.57^c^ ± 6.82	47.45^a^ ± 7.29	84.91^c^ ± 8.61	6.76^a^ ± 0.02	6.76^a^ ± 0.02	−54.95^ab^ ± 6.17	−60.05^a^ ± 1.10

UST-400MPa-70 °C	Raw milk	3.53^p^* ± 0.90	7.55^p^* ± 0.69	16.66^p^* ± 3.03	18.97^p^* ± 4.79	6.73^p^ ± 0.03	6.74^p^ ± 0.03	−35.92^p^* ± 0.36	−37.56^pq^* ± 0.83
	Skim milk	2.92^p^* ± 0.08	8.30^p^* ± 1.55	15.29^p^ ± 1.64	15.34^p^* ± 3.54	6.77^p^ ± 0.03	6.78^p^ ± 0.03	−36.03^pq^* ± 1.00	−36.35^pq^* ± 0.63
	Cream	46.81^q^* ± 6.47	77.20^r^* ± 5.48	49.97^q^ ± 5.97	51.05^q^* ± 0.58	6.78^p^ ± 0.03	6.80^p^ ± 0.04	−45.20^r^ ± 3.81	−40.69^qr^* ± 1.02

a Results are expressed as Mean ± Standard Deviation.

b For comparisons within the Untreated group, means with the same superscripts (a–c) are not significantly different. For comparisons within the UST treated group, means with the same superscripts (p–r) are not significantly different. Mean values for UST treated samples that were significantly different from Untreated samples of the same fat level and pea protein inclusion are denoted by an asterisk.

**Table 3:** Significance levels (P) of the three-way analysis of variance for the effects of fat level, pea protein and UST treatment.

	Mean diameter (μm)	Average height (μm)	pH	Zeta potential (mV)
Fat level	***	***	***	***
Pea protein	***	***	*	NS
Treatment	***	***	***	***
Fat level × Pea protein	NS	**	NS	NS
Fat level × Treatment	***	***	NS	NS
Pea protein × Treatment	***	***	NS	NS
Fat level × Pea protein × Treatment	***	**	NS	**

NS - Not Significant, *P ≤ 0.05, **P ≤ 0.01, ***P ≤ 0.001.

On the contrary, for high-fat samples, i.e. cream without and with pea protein, the UST treatment increased the mean particle diameter (Table 2). The average height of UST-treated cream and cream + pea protein samples were significantly higher (*P* < 0.05) than other UST-treated samples. In these samples, UST could reduce the particle size of fat globules and protein aggregates. However, the collisions between densely placed fat and other components could in turn facilitate aggregation among smaller fat globules, casein neo-micelles, whey protein, and pea proteins to create a colloidal matrix with apparently larger particle sizes.

This finding reinforces that the high pressure and thermal effects during UST could cause partial protein unfolding and lead to the protein association with densely placed fat and other components, thus increasing the particle size in the cream + pea protein dispersions. This particle size increase could also be responsible for higher gel firmness (Dumay et al., 2013).

##### 3.3.2.1 Soluble protein analysis

Within the experimental conditions of the study, the UST treatment increased (*P* < 0.05) the soluble protein content and the solubilities of the dairy-pea protein dispersions (Table 4). In our previous study, the protein solubilities of milk-pea dispersions with varying pea protein concentration from 1:0.5 to 1:3 with initial solubilities of 17.27–28.23% increased significantly (*P* < 0.05) from 31.28 to 34.47% after 70 °C UST treatment (Janahar et al., 2022). The solubility increase could be contributed by the particle size reduction caused by high pressure-associated shear. This increase is in accordance with observations by Krentz et al. (2022), who reported increase in soluble protein content in milk-pea blends (casein:pea protein 1:1 protein ratio) combined at 50:50 ratio, after shear treatment in a two stage homogenizer at 24 MPa (first stage) and 3 MPa (second stage) at 4 °C. The high shear disrupts the 3-dimensional globular pea proteins into smaller and soluble aggregates, which resulted in increased soluble protein content and solubility (Krentz et al., 2022).

**Table 4:** Soluble protein parameters of dispersed samples^a^.

S.No.	Sample^b^	Water content (%)	Fat (%)	Untreated			UST-400MPa-70 °C		
				Soluble protein (mg/mL)	Solubility (%)	Hydrodynamic diameter (nm)	Soluble protein (mg/mL)	Solubility (%)	Hydrodynamic diameter (nm)
1	Raw milk + Pea protein	82.76 ± 0.45	5.31 ± 0.38	45.60^a^ ± 1.80	61.23^a^ ± 5.00	221.89^a^ ± 0.58	50.36^a^* ± 1.01	70.19^a^* ± 1.31	676.09^a^* ± 10.11
2	Skim milk + Pea protein	86.81 ± 0.09	0.35 ± 0.02	44.40^a^ ± 2.06	61.53^a^ ± 4.24	185.21^b^ ± 2.28	54.29^b^* ± 2.53	76.98^a^* ± 0.15	245.62^b^* ± 4.68
3	Cream + Pea protein	48.84 ± 0.15	50.39 ± 0.10	22.74^b^ ± 3.93	32.85^b^ ± 4.14	262.32^c^ ± 4.49	33.57^c^* ± 1.52	49.02^b^* ± 5.23	324.04^c^* ± 6.70

a Results are expressed as Mean ± Standard Deviation.

b Within each treatment group (Untreated and UST), means in same column with same superscripts (a-c) are not significantly different (*P* ≤ 0.05). Mean values for UST treated samples that were significantly different from Untreated samples in same row are denoted by an asterisk.

Furthermore, the soluble protein concentration and solubilities varied according to the level of fat (Table 4). The solubilities of untreated and UST-treated raw milk + pea protein and skim milk + pea protein dispersions were higher (*P* < 0.05) than cream + pea protein samples. This difference could be attributed to the fat and water contents in the dispersions. For instance, presence of more water content in skim milk dispersion could be responsible for the higher soluble protein in these samples after UST. The particle breakdown caused by UST and concurrent availability of more water and presence of less fat could help solubilizing the particles. The low protein solubility in cream + pea protein samples could be responsible for higher consistency, since it has been reported that insoluble proteins have high fat binding capacity (Zayas, 1997).

The hydrodynamic diameter of the particles in the aqueous phase of all the dispersions increased significantly (*P* < 0.05) after UST treatment. In untreated dispersions, the insoluble pea proteins settled down as pellets. In UST-treated dispersions, the pea proteins ruptured by pressure-associated shear might have associated with other pea proteins, milk proteins, or fat in the supernatant to increase the hydrodynamic diameter. This change demonstrated the role of UST in protein solubility and the particle size modification achieved by UST through inter-particle interactions.

### 3.4 pH and zeta potential

The pH and zeta potential of samples are given in Table 2. The interaction of UST treatment, pea protein inclusion and fat level did not have significant influence on the pH of the samples (Table 3). The pH of samples of different fat levels treated by UST were not significantly different from untreated samples, in accordance with earlier research (Pereda et al., 2007; Janahar et al., 2021). pH contributes to modification of dissociation or association between protein subunits and so the adsorption of pea protein at the fat–water interface is dependent on pH (Gharsallaoui et al., 2009). Thus, the change in the rheological properties of UST-treated dispersions with pea proteins in the present study could not be attributed to change in pH. No change in pH indicates that the gel formation in UST-treated high-fat samples could not be due to coagulation of milk proteins caused by lactic acid production.

The zeta potential indicates the magnitude of charge on a colloidal particle. The UST treatment showed significant effect (*P* ≤ 0.001) on zeta potential. For all samples, the zeta potential reduced after UST treatment (Table 2). The pressure-associated shear action in UST treatment could cause surface modifications in proteins and make the charged amino acid residues of the proteins move from the surface to the interior or create protein–protein linkage, thereby masking negative charges (Relkin and Shukat, 2012; Janahar et al., 2021). Wang et al. (2011) reported reduction of zeta potential in flaxseed gum solutions processed at homogenization pressures above 10 MPa. The authors attributed the reduction in molecule chain size of flaxseed gum by HPH at pressures higher than 10 MPa to be responsible for reduction in zeta potential. Thus, the variation in zeta potential showed the effect of UST in changing structural characteristics of the dairy-pea protein blends.

### 3.5 Stability

**Fig. 6:**
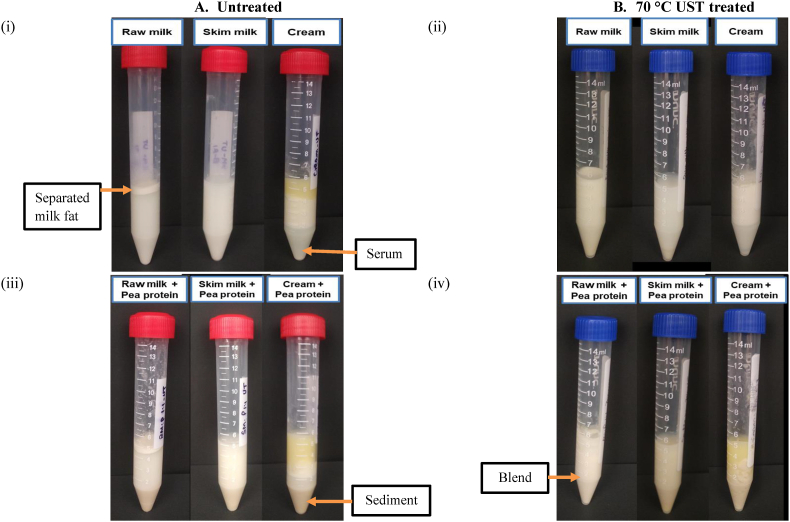
Macroscopic observations of dispersions (A: before and B: after UST treatment) heat treated at ∼100 °C for 10 min and centrifuged at 4000×*g* for 30 min at 4 °C.

The stability of the dairy-pea protein dispersions against creaming or sedimentation was determined through accelerated separation by centrifugation, followed by visual observation of the different fractions of separated milk fat, sediment, serum, and blend. A representative macroscopic analysis of different fractions in the samples is shown in Fig. 6.

#### 3.5.1 Colloidal stability

**Fig. 7:**
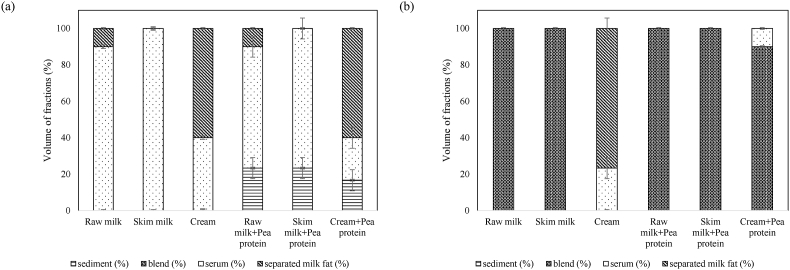
Volume (%) of different fractions in (a) untreated and (b) 70 °C UST-treated samples after centrifugation for 30 min at 4000×*g* (4 °C).

The volumes of different fractions that separated after centrifugation of samples are shown in Fig. 7. In general, higher amounts of separated milk fat and pea protein are indicators of lower stability. After centrifugation, the untreated raw milk and cream samples showed a separated layer of milk fat on top due to the natural phenomenon of clustering and rising of fat globules. Visually, all the untreated samples with added pea protein showed clear sedimentation of pea proteins at the bottom, thereby showing the inability of the milk matrix to self-stabilize by keeping the pea protein particles suspended in dispersions. Conversely, all UST-treated samples were free from sediments and creaming phenomena. The lack of sediments and creaming demonstrated the ability of the UST process to create a dairy-pea protein blended colloid and keep it stable during refrigerated storage. In low-fat concentration samples, i.e., raw milk, skim milk, raw milk + pea protein, and skim milk + pea protein samples, the stability could be mainly contributed by the reduction of particle size. In the high-fat concentration samples, the stability could be contributed by the high viscosity of the samples in accordance with Stoke's law. Interestingly, the serum volume in untreated cream samples is significantly (*P* < 0.05) higher than UST-treated samples. The effect on UST in creating fat and protein networks to entrap more serum is thus realized. Similarly, the volume of serum in untreated cream + pea protein was significantly (*P* < 0.05) higher than UST-treated cream + pea protein samples, which indicated higher water-binding capacity in treated samples and the possible relation to consistency of the samples, as discussed earlier in section 3.2.4.

#### 3.5.2 Heat stability

**Fig. 8:**
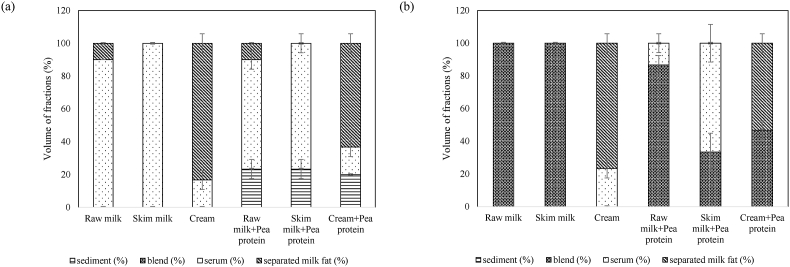
Volume (%) of different fractions in (a) untreated and (b) 70 °C UST-treated samples heat treated at ∼100 °C for 10 min followed by centrifugation for 30 min at 4000×*g* (4 °C).

The volume fractions of different layers in samples after heat treatment were not significantly different from the samples before heat treatment. Thus, the UST-treated samples were stable to heat treatment at the conditions tested. However, centrifugation of the samples after heat treatment led to separation of fractions (Fig. 8). For instance, the UST-treated raw milk + pea protein and skim milk + pea protein blends showed separation of the liquid serum from the blends. This separation could be due to the reduced water retention capacity of the blends, due to whey protein denaturation caused by heat treatment (Khalesi and FitzGerald, 2021). Lee (1992) reported that at temperatures >70 °C, intermolecular disulfide bonds could form in the protein molecules and parts of the peptide chain could link with each other by hydrophobic interactions. Consequently, protein aggregation and precipitation could occur. Separation of fat from the blend by melting during heat treatment in UST-treated cream + pea protein samples is worth noting (Fig. 8, Fig. 6-iv). Hence, though the UST-treated dairy-pea samples appear stable immediately after heat treatment, an eventual onset of separation of contents could be expected.

#### 3.5.3 Freeze–thaw stability

**Fig. 9:**
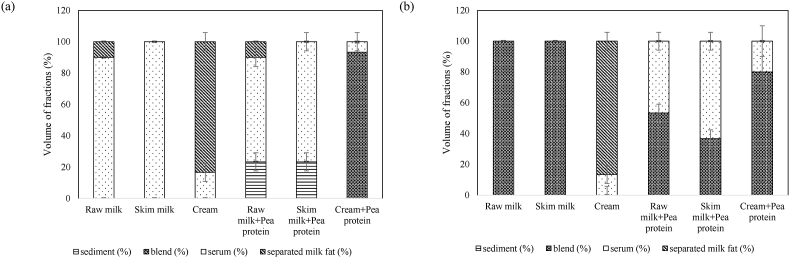
Volume (%) of different fractions in (a) untreated and (b) 70 °C UST-treated samples under freeze (−20 °C/24 h)–thaw (4 °C/12 h) treatment followed by centrifugation for 30 min at 4000×*g* (4 °C).

All 70 °C UST samples were stable after freeze–thaw treatment and showed no separation of their components. However, subsequent centrifugation of samples showed liquid serum separation in UST-treated dairy-pea protein blended samples (Fig. 9). The serum is separated from the blend of pea protein and fat after centrifugation. Additionally, the volume of the blend corresponds to the amount of protein and fat in the dispersion (Fig. 9). As discussed earlier, the UST treatment facilitates association of protein and fat in the dispersions and enhance the water-binding capacity. During freezing, the frozen water leads to freeze-concentration of solids and reorganization of the molecules (Zheng and Sosulski, 1998). Subsequently, ice crystals could cause disruption of networks resulting in release of entrapped water during thawing. This disruption could have led to liquid serum separation in UST-treated samples after freeze–thaw treatment and subsequent centrifugation.

## 4 Conclusions

Our study demonstrated that suitable combination of UST process parameters (pressure, temperature, shear) and product formulation (pea, dairy protein, and fat level) can be used to produce dispersions of a wide range of rheological and functional characteristics. The interaction of fat level, pea protein and UST had significant effect on the mean particle diameter of the dispersions. The mean diameter of low-fat (skim milk + pea protein) dispersions decreased from 51.76 μm to 8.30 μm, whereas that of high-fat (cream + pea protein) dispersion increased from 48.57 μm to 77.20 μm after UST treatment. UST treatment increased the consistency coefficient of dispersions with increase in fat level. For instance, the consistency coefficient of raw milk + pea protein, and skim milk + pea protein dispersions increased from 0.42 to 1.37 Pa·s^*n*^ (226% increase) and 0.09–0.14 Pa·s^*n*^ (56% increase) after UST treatment. The soluble protein contents and solubilities of dispersions with pea protein increased by UST treatment. The accelerated stability testing by centrifugation showed that the UST-treated samples were stable with no sedimentation or creaming. Thus, UST can be used for formulating stable dispersions without the need for any synthetic emulsifier or stabilizers. The knowledge obtained in the present study will facilitate process development of novel clean-label dairy-plant protein dispersions and design the UST equipment.

## CRediT authorship contribution statement

**Jerish Joyner Janahar:** Conceptualization, Methodology, Investigation, Formal analysis, Data curation, Writing – original draft. **V.M. Balasubramaniam:** Conceptualization, Resources, Methodology, Formal analysis, Supervision, Project administration, Funding acquisition, Writing – review & editing. **Rafael Jiménez-Flores:** Conceptualization, Methodology, Writing – review & editing, Supervision, Funding acquisition. **Osvaldo H. Campanella:** Writing – review & editing, Methodology, Formal analysis, Writing – review & editing. **Bhavesh Patel:** Methodology, Formal analysis, Data curation, Writing – review & editing. **Joana Ortega-Anaya:** Methodology, Writing – review & editing.

## Declaration of competing interest

The authors declare the following financial interests/personal relationships which may be considered as potential competing interests: VM Balasubramaniam reports the research was sponsored by 10.13039/100005825USDA National Institute of Food and Agriculture grant 2018-67017-27914.

## Data Availability

Data will be made available on request.

## References

[bib1] Aguilar-Zárate M., De la Peña-Gil A., Álvarez-Mitre F.M., Charó-Alonso M.A., Toro-Vazquez J.F. (2019). Vegetable and mineral oil organogels based on monoglyceride and lecithin mixtures. Food Biophys..

[bib2] Akhtar M., Stenzel J., Murray B., Dickinson E. (2005). Factors affecting the perception of creaminess of oil-in-water emulsions. Food Hydrocolloids.

[bib3] Baier D., Schmitt C., Knorr D. (2015). Changes in functionality of whey protein and micellar casein after high pressure – low temperature treatments. Food Hydrocolloids.

[bib4] Balasubramaniam V.M. (2021). Process development of high pressure-based technologies for food: research advances and future perspectives. Curr. Opin. Food Sci..

[bib5] Balasubramaniam, V.M., Lee, J., Serventi, L., 2023. Understanding new foods: development of next generation of food processing, packaging, and ingredients technologies for clean-label foods. Chapter 12 In: Serventi, L. (Ed.), Sustainable Food Innovation, Sustainable Development Goals Series. Springer Cham. Switzerland (in press).

[bib6] Barrantes E., Tamime A.Y., Sword A.M., Muir D.D., Kalab M. (1996). The manufacture of set-type natural yoghurt containing different oils—2: rheological properties and microstructure. Int. Dairy J..

[bib7] Boukid F., Rosell C.M., Castellari M. (2021). Pea protein ingredients: a mainstream ingredient to (re)formulate innovative foods and beverages. Trends Food Sci. Technol..

[bib8] Chojnicka-Paszun A., de Jongh H.H.J., de Kruif C.G. (2012). Sensory perception and lubrication properties of milk: influence of fat content. Int. Dairy J..

[bib9] Cortés-Muñoz M., Chevalier-Lucia D., Dumay E. (2009). Characteristics of submicron emulsions prepared by ultra-high pressure homogenisation: effect of chilled or frozen storage. Food Hydrocolloids.

[bib10] Dumay E., Chevalier-Lucia D., Picart-Palmade L., Benzaria A., Gràcia-Julià A., Blayo C. (2013). Technological aspects and potential applications of (ultra) high-pressure homogenisation. Trends Food Sci. Technol..

[bib11] Ferragut V., Cruz N.S., Trujillo A., Guamis B., Capellas M. (2009). Physical characteristics during storage of soy yogurt made from ultra-high pressure homogenized soymilk. J. Food Eng..

[bib12] Floury J., Desrumaux A., Legrand J. (2002). Effect of ultra-high-pressure homogenization on structure and on rheological properties of soy protein-stabilized emulsions. J. Food Sci..

[bib13] Floury J., Legrand J., Desrumaux A. (2004). Analysis of a new type of high pressure homogeniser. Part B. study of droplet break-up and recoalescence phenomena. Chem. Eng. Sci..

[bib14] García-Ortega M.L., Toro-Vazquez J.F., Ghosh S. (2021). Development and characterization of structured water-in-oil emulsions with ethyl cellulose oleogels. Food Res. Int..

[bib15] Gharsallaoui A., Cases E., Chambin O., Saurel R. (2009). Interfacial and emulsifying characteristics of acid-treated pea protein. Food Biophys..

[bib16] Hansen S.F., Larsen L.B., Wiking L. (2019). Thermal effects on IgM-milk fat globule-mediated agglutination. J. Dairy Res..

[bib17] Hayes M.G., Kelly A.L. (2003). High pressure homogenisation of raw whole bovine milk (a) effects on fat globule size and other properties. J. Dairy Res..

[bib18] Hinderink E.B.A., Münch K., Sagis L., Schroën K., Berton-Carabin C.C. (2019). Synergistic stabilisation of emulsions by blends of dairy and soluble pea proteins: contribution of the interfacial composition. Food Hydrocolloids.

[bib19] Ho K.K.H.Y., Schroën K., San Martín-González M.F., Berton-Carabin C.C. (2018). Synergistic and antagonistic effects of plant and dairy protein blends on the physicochemical stability of lycopene-loaded emulsions. Food Hydrocolloids.

[bib20] Janahar J.J., Balasubramaniam V.M., Jimenez-Flores R., Campanella O.H., García-Cano I., Chen D. (2022). Pressure, shear, thermal, and interaction effects on quality attributes of pea–dairy protein colloidal dispersions. Food Hydrocolloids.

[bib21] Janahar J.J., Marciniak A., Balasubramaniam V.M., Jimenez-Flores R., Ting E. (2021). Effects of pressure, shear, temperature, and their interactions on selected milk quality attributes. J. Dairy Sci..

[bib22] Jervis S.M., Gerard P., Drake S., Lopetcharat K., Drake M.A. (2014). The perception of creaminess in sour cream. J. Sensory Stud..

[bib23] Ji J., Zhang J., Chen J., Wang Y., Dong N., Hu C., Chen H., Li G., Pan X., Wu C. (2015). Preparation and stabilization of emulsions stabilized by mixed sodium caseinate and soy protein isolate. Food Hydrocolloids.

[bib24] Khalesi M., FitzGerald R.J. (2021). Physicochemical properties and water interactions of milk protein concentrate with two different levels of undenatured whey protein. Colloids Surf. A Physicochem. Eng. Asp..

[bib25] Krentz A., García-Cano I., Ortega-Anaya J., Jiménez-Flores R. (2022). Use of casein micelles to improve the solubility of hydrophobic pea proteins in aqueous solutions via low-temperature homogenization. J. Dairy Sci..

[bib26] Lam A.C.Y., Can Karaca A., Tyler R.T., Nickerson M.T. (2018). Pea protein isolates: structure, extraction, and functionality. Food Rev. Int..

[bib27] Lee Y.-H. (1992). Food-processing approaches to altering allergenic potential of milk-based formula. J. Pediatr..

[bib28] Li Y., Joyner H.S., Carter B.G., Drake M.A. (2018). Effects of fat content, pasteurization method, homogenization pressure, and storage time on the mechanical and sensory properties of bovine milk. J. Dairy Sci..

[bib29] Marcotte M., Taherian Hoshahili A.R., Ramaswamy H.S. (2001). Rheological properties of selected hydrocolloids as a function of concentration and temperature. Food Res. Int..

[bib30] Martínez-Monteagudo S.I., Kamat S., Patel N., Konuklar G., Rangavajla N., Balasubramaniam V.M. (2017). Improvements in emulsion stability of dairy beverages treated by high pressure homogenization: a pilot-scale feasibility study. J. Food Eng..

[bib31] McCarthy K.S., Lopetcharat K., Drake M.A. (2017). Milk fat threshold determination and the effect of milk fat content on consumer preference for fluid milk. J. Dairy Sci..

[bib32] Mession J.-L., Sok N., Assifaoui A., Saurel R. (2013). Thermal denaturation of pea globulins (*Pisum sativum* L.)—molecular interactions leading to heat-induced protein aggregation. J. Agric. Food Chem..

[bib33] Morales Francisco-J., Romero C., Jimenez-Perez S. (2000). Characterization of industrial processed milk by analysis of heat-induced changes. Int. J. Food Sci. Technol..

[bib34] Pereda J., Ferragut V., Quevedo J.M., Guamis B., Trujillo A.J. (2007). Effects of ultra-high pressure homogenization on microbial and physicochemical shelf life of milk. J. Dairy Sci..

[bib35] Relkin P., Shukat R. (2012). Food protein aggregates as vitamin-matrix carriers: impact of processing conditions. Food Chem..

[bib36] Roach A., Harte F. (2008). Disruption and sedimentation of casein micelles and casein micelle isolates under high-pressure homogenization. Innovat. Food Sci. Emerg. Technol..

[bib37] Rønholt S., Mortensen K., Knudsen J.C. (2013). The effective factors on the structure of butter and other milk fat-based products. Compr. Rev. Food Sci. Food Saf..

[bib38] Serra M., Trujillo A.J., Guamis B., Ferragut V. (2009). Evaluation of physical properties during storage of set and stirred yogurts made from ultra-high pressure homogenization-treated milk. Food Hydrocolloids.

[bib39] Smejkal G.B., Ting E.Y., Nambi Arul Nambi K., Schumacher R.T., Lazarev A.V. (2021). Characterization of astaxanthin nanoemulsions produced by intense fluid shear through a self-throttling nanometer range annular orifice valve-based high-pressure homogenizer. mol.

[bib40] Smith P.K., Krohn R.I., Hermanson G.T., Mallia A.K., Gartner F.H., Provenzano M.D., Fujimoto E.K., Goeke N.M., Olson B.J., Klenk D.C. (1985). Measurement of protein using bicinchoninic acid. Anal. Biochem..

[bib41] Song X., Zhou C., Fu F., Chen Z., Wu Q. (2013). Effect of high-pressure homogenization on particle size and film properties of soy protein isolate. Ind. Crop. Prod..

[bib42] Tanger C., Utz F., Spaccasassi A., Kreissl J., Dombrowski J., Dawid C., Kulozik U. (2021). Influence of pea and potato protein microparticles on texture and sensory properties in a fat-reduced model milk dessert. ACS Food Sci. Technol..

[bib43] Tran M., Roberts R., Felix T.L., Harte F.M. (2018). Effect of high-pressure-jet processing on the viscosity and foaming properties of pasteurized whole milk. J. Dairy Sci..

[bib44] Utsumi Shigeru, Matsumura Y., Mori T., Damodaran S., Paraf A. (2017). Food Proteins and Their Applications.

[bib45] Vélez‐Ruiz J.F., Barbosa Cánovas G.V., Peleg M. (1997). Rheological properties of selected dairy products. Crit. Rev. Food Sci. Nutr..

[bib46] Vliet T., Walstra P. (1980). Relationship between viscosity and fat content of milk and cream. J. Texture Stud..

[bib47] Wang Y., Li D., Wang L.-J., Xue J. (2011). Effects of high pressure homogenization on rheological properties of flaxseed gum. Carbohydr. Polym..

[bib48] Xiu A., Zhou M., Zhu B., Wang S., Zhang J. (2011). Rheological properties of Salecan as a new source of thickening agent. Food Hydrocolloids.

[bib49] Xu Z.M., Emmanouelidou D.G., Raphaelides S.N., Antoniou K.D. (2008). Effects of heating temperature and fat content on the structure development of set yogurt. J. Food Eng..

[bib50] Zamora A., Ferragut V., Guamis B., Trujillo A.J. (2012). Changes in the surface protein of the fat globules during ultra-high pressure homogenisation and conventional treatments of milk. Food Hydrocolloids.

[bib51] Zamora A., Ferragut V., Juan B., Guamis B., Trujillo A.J. (2011). Effect of ultra-high pressure homogenisation of milk on the texture and water-typology of a starter-free fresh cheese. Innovat. Food Sci. Emerg. Technol..

[bib52] Zayas J.F. (1997). Functionality of Proteins in Food.

[bib53] Zheng G.H., Sosulski F.W. (1998). Determination of water separation from cooked starch and flour pastes after refrigeration and freeze-thaw. J. Food Sci..

